# Reticulate Evolutionary History of a Complex Group of Grasses: Phylogeny of *Elymus* StStHH Allotetraploids Based on Three Nuclear Genes

**DOI:** 10.1371/journal.pone.0010989

**Published:** 2010-06-09

**Authors:** Roberta J. Mason-Gamer, Melissa M. Burns, Marianna Naum

**Affiliations:** Department of Biological Sciences, The University of Illinois at Chicago, Chicago, Illinois, United States of America; Montreal Botanical Garden, Canada

## Abstract

**Background:**

*Elymus* (Poaceae) is a large genus of polyploid species in the wheat tribe Triticeae. It is polyphyletic, exhibiting many distinct allopolyploid genome combinations, and its history might be further complicated by introgression and lineage sorting. We focus on a subset of *Elymus* species with a tetraploid genome complement derived from *Pseudoroegneria* (genome **St**) and *Hordeum* (**H**). We confirm the species' allopolyploidy, identify possible genome donors, and pinpoint instances of apparent introgression or incomplete lineage sorting.

**Methodology/Principal Findings:**

We sequenced portions of three unlinked nuclear genes—phosphoenolpyruvate carboxylase, β-amylase, and granule-bound starch synthase I—from 27 individuals, representing 14 Eurasian and North American **StStHH** *Elymus* species. *Elymus* sequences were combined with existing data from monogenomic representatives of the tribe, and gene trees were estimated separately for each data set using maximum likelihood. Trees were examined for evidence of allopolyploidy and additional reticulate patterns. All trees confirm the **StStHH** genome configuration of the *Elymus* species. They suggest that the **StStHH** group originated in North America, and do not support separate North American and European origins. Our results point to North American *Pseudoroegneria* and *Hordeum* species as potential genome donors to *Elymus*. Diploid *P. spicata* is a prospective **St-**genome donor, though conflict among trees involving *P. spicata* and the Eurasian *P. strigosa* suggests either introgression of GBSSI sequences from *P. strigosa* into North American *Elymus* and *Pseudoroegneria*, or incomplete lineage sorting of ancestral GBSSI polymorphism. Diploid *H. californicum* and/or allotetraploid *H. jubatum* are possible **H**-genome donors; direct involvement of an allotetraploid *Hordeum* species would simultaneously introduce two distinct **H** genomes to *Elymus*, consistent with some of the relationships among **H**-genome sequences in *Hordeum* and *Elymus*.

**Conclusions/Significance:**

Comparisons among molecular phylogenetic trees confirm allopolyploidy, identify potential genome donors, and highlight cases of apparent introgression or incomplete lineage sorting. The complicated history of this group emphasizes an inherent problem with interpreting conflicts among bifurcating trees—identifying introgression and determining its direction depend on which tree is chosen as a starting point of comparison. In spite of difficulties with interpretation, differences among gene trees allow us to identify reticulate species and develop hypotheses about underlying evolutionary processes.

## Introduction

Untangling reticulate relationships among species presents an interesting challenge to systematists, and an opportunity to uncover previously undetected evolutionary processes. Comparisons among gene trees can clarify historical relationships among species, and the examination of topological conflicts among trees can reveal complicating factors such as retention of ancestral genetic polymorphism, past or ongoing gene exchange, allopolyploidy, or a combination of these. Distinguishing among potential causes of phylogenetic conflict is often difficult, but careful comparisons among trees can help pinpoint the species involved, and allow specific hypotheses to be formulated. In the present study, we focus on species that are explicitly reticulate in that they are all allotetraploids, and potentially secondarily reticulate if they have arisen through multiple independent origins or undergone hybridization at the tetraploid level. We assess the role of reticulation at both levels in the genus *Elymus* L. of the wheat tribe Triticeae (Poaceae), using phylogenetic analyses of three unlinked, low-copy genetic markers.

Many inferences of reticulate evolution have been based on comparisons among gene trees; in plants, comparisons between chloroplast DNA (cpDNA) and nuclear ribosomal internal transcribed spacer (ITS) phylogenies are especially widely used (e.g., [1]–[7]). The reasons are partly historical – for technical reasons, these high-copy markers were among the first to be widely used for plant phylogenetic studies. They remain in frequent use in studies of reticulation (e.g., [8]–[12]), where they offer both methodological advantages and a kingdom-wide backdrop of published sequences within which new data can be interpreted. However, both cpDNA and ITS sequence data sets have some disadvantages. The chloroplast genome is maternally inherited in most angiosperms, and its ability to identify a maternal donor can be an advantage. However, its inability to provide information about other genetic donors is often a major limitation. The biparentally-inherited ITS does have the potential to reveal multiple genome donors, but its arrangement in long repetitive arrays promotes the confounding effects of concerted evolution, both within arrays [13]–[15] and among them [16], [17]. Thus, ITS copies can potentially convert toward one or the other parent, and the resulting sequence homogeneity can obscure a history of contributions from multiple distinct donors.

Low-copy nuclear genes can, like ITS, reveal multiple genome donors, and they are less subject to gene conversion. However, they do have some practical disadvantages. They can be more difficult to amplify because of their low copy number, and because online databases often contain fewer comparable sequences from which amplification primers can be designed. The smaller sequence database also narrows the phylogenetic context within which new data sets can be analyzed, and makes it more difficult to assemble the crucial copy-number information that would prevent misinterpretation of unsuspected variation among paralogs. In spite of the difficulties, a variety of single- and low-copy nuclear genes have been successfully used in many studies of reticulate relationships in plants over the last decade (e.g., [18]–[32]). Sequence data from some low-copy genes are now becoming plentiful across a broad range of angiosperms.

This study presents three low-copy nuclear gene trees from a group of tetraploid species in the wheat tribe, Triticeae. The wheat tribe is especially well known for its economically important members, including wheat, barley, and rye. The tribe's economic importance has driven an interest in its evolutionary history seemingly disproportionate to its size (about 300 species), yet a singular, straightforward phylogenetic estimate for the tribe remains elusive. One reason for this is that a history of incomplete lineage sorting and/or gene exchange has complicated relationships among the diploid lineages, so that sequence data from different genes yield conflicting trees [33], [34]. A second confounding issue is that the tribe includes a large number of genetically diverse allopolyploid lineages. The most well known of these are the tetraploid and hexaploid cultivated wheats (*Triticum* L.), but far more numerous are those that combine genomes from the wheatgrass genus *Pseudoroegneria* (Nevski) Á.Löve (genome designation **St**) with one or more genomes from other Triticeae genera (e.g., [35]). Under the genomic definition of genera [35], [36] widely applied to the Triticeae, most of the **St**-genome allopolyploids are classified as *Elymus*. Within *Elymus*, the **St** genome can be combined with a variety of other genomes, including that of *Hordeum* L. (genome designation **H**), *Agropyron* Gaertn. (**P**), *Australopyrum* (Tzvelev) Á.Löve (**W**), and an unknown donor (**Y**), and in many combinations including **StStHH**, **StStYY**, **StStHHHH**, **StStStStHH**, **StStStStYY**, **StStYYYY**, **StStHHYY**, **StStYYWW**, and **StStYYPP** [36]–[49]. Other **St**-containing allopolyploids include the autoallooctoploid *Pascopyrum smithii* (Rydb.) Á. Löve, which combines the *Pseudoroegneria* and *Hordeum* genomes with the **Ns** genome of *Psathyrostachys* Nevski an **StStHHNsNsNsNs** configuration [50]. *Thinopyrum* Á.Löve includes some octo- and decaploid species which are hypothesized to combine the **St** genome with the **E** and/or **J** genomes characteristic of *Thinopyrum* [51]–[53].

In this study, we focus on the **StStHH** *Elymus* tetraploids. This northern temperate group of about 50 species is distributed throughout much of North America, Europe, and western Asia. Numerous studies provide evidence that *Pseudoroegneria* and *Hordeum* were the genome donors to these tetraploids (e.g., [54] and references therein). Our results clearly confirm these studies, but we considered the **StStHH** genome configuration to be well-established before the start of the study, rather than a hypothesis in need of additional tests. Our main goals, therefore, were to: (1) determine whether the North American vs. Eurasian **StStHH** species arose from separate polyploidization events; (2) identify possible progenitor species from within *Hordeum* and *Pseudoroegneria*; and (3) find out whether reticulate patterns in **StStHH** *Elymus* extend beyond those clearly attributable to allopolyploidy alone.

## Materials and Methods

### Samples

The analyses include 27 individuals representing 14 **StStHH** tetraploid *Elymus* species: 11 individuals representing five Eurasian species (Table 1), and 16 individuals representing nine North American species (Table 2). Most of the accessions were obtained from the USDA and have associated chromosome counts (2n = 28), confirming their tetraploid nature. Nearly all of the sequences from the Eurasian *Elymus* samples were newly generated for this study; the few exceptions [55] are noted in Table 1. Many of the North American *Elymus* pepC and GBSSI sequences were drawn from earlier studies [29], [56], while the North American β-amylase sequences are new. The numbers of intraspecific samples of North American species differ among the three gene trees, but this does not affect our main conclusions.

**Table 1:** Eurasian **StStHH** tetraploid *Elymus* species.

	Accession^1^	Country of Origin	#^2^	Genome	pepC	b-amylase	GBSSI^5^
*Elymus brachyaristatus*	PI 499411	China	1	**St**	b^3^-HM035290	b-HM035223	a-HM035268
				**H**	e-HM035291	a-HM035224	b-HM035269
*Elymus caninus*	PI 314205	Uzbekistan	1	**St**	h-HM035292	a-HM035225	n-DQ159325
				**H**	b-HM035293	d-HM035226	a-DQ159324
	PI 314612	Kazakhstan	2	**St**	a-HM035294	d-HM035227	b-HM035270
				**H**	d-HM035295	c-HM035228	a-HM035271
					i-HM035296		
	PI 499413	China	4	**St**	d-HM035297	d-HM035229	a-HM035272
				**H**	f-HM035298	b-HM035230	b-HM035273
	PI 531571	Poland	5	**St**	e-HM035299	d-HM035231	a-HM035274
				**H**	g-HM035300	e-HM035232	b-HM035275
*Elymus dentatus*	PI 628702	Russia	1	**St**	d-HM035301	a-HM035233	a-DQ159328
				**H**	c-HM035302	d-HM035234	b-DQ159329
					e-HM035303		
	PI 531599	Pakistan	2	**St**	a-HM035304	i-HM035235	a-HM035276
				**H**	b-HM035305	a-HM035236	b-HM035277
*Elymus mutabilis*	PI 628704	Russia	1	**St**	NR^4^	a-HM035237	c-DQ159331
				**H**	a-HM035306	c-HM035238	a-DQ159330
					i-HM035307		
	PI 499449	China	2	**St**	h-HM035308	h-HM035239	a-HM035278
				**H**	a-HM035309	c-HM035240	b-HM035279
					f-HM035310		
*Elymus sibiricus*	PI 628699	Russia	1	**St**	b-HM035311	NR	b-HM035280
				**H**	a-HM035312	b-HM035241	a-HM035281
	PI 499461	China	3	**St**	d-HM035313	g-HM035242	a-HM035282
				**H**	a-HM035314	b-HM035243	b-HM035283

1 Plant introduction (PI) numbers were assigned by the USDA. Voucher specimens are at ID.

**Figure 1:**
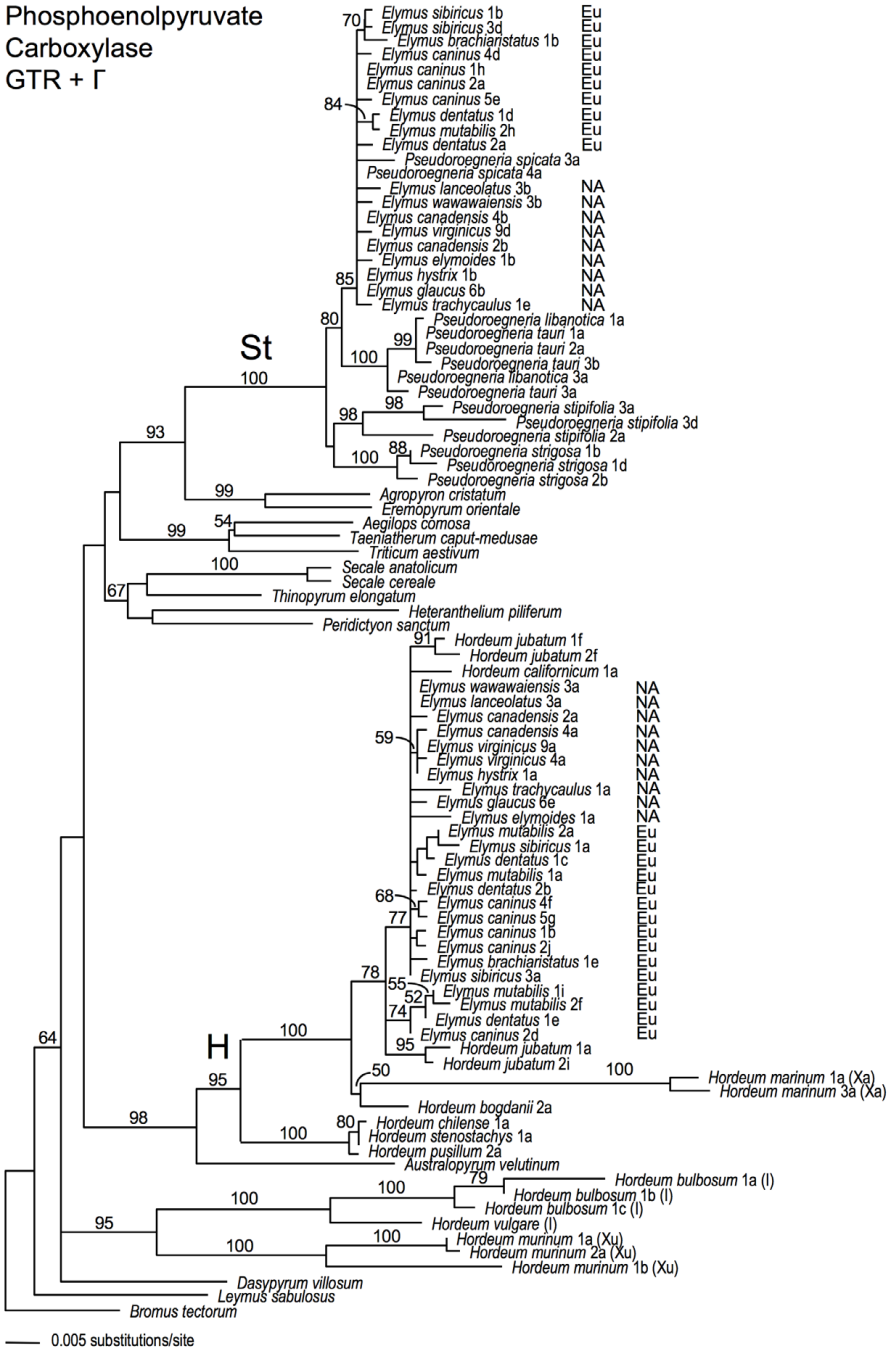
Phosphoenolpyruvate carboxylase gene tree. The best-scoring ML tree was selected from 30 GARLI analyses of the pepC sequence data set under a GTR+Γ model of sequence evolution. Numbers above branches show ML bootstrap support ≥50%. “NA” and “Eu” distinguish North American and Eurasian *Elymus* species, respectively. Where applicable, numbers following taxon names distinguish individuals within species, and are consistent among Figures 1–3, S1. Letters following these numbers designate cloned sequences from within individuals, and are specific to each gene tree.

2 Numbers (#) distinguish individuals within species on Figures 1–3, S1.

3 Letters identify cloned sequences from within each individual on Figures 1–3, S1.

4 NR: not recovered for a gene/individual.

5 DQ1593## accessions: [55]; all others are new.

**Table 2:** North American **StStHH** tetraploid *Elymus* species.

	Accession^1^	#^2^	Genome	pepC^6^	β-amylase	GBSSI^7^
*Elymus canadensis*	PI 578675	2	**St**	b^3^-HM035315	-4	-
			**H**	a^3^-AY553243	-	-
*Elymus canadensis*	PI 531568	4	**St**	b-AY553248	a-HM035244	c-HM035284
			**H**	a-AY553242	d-HM035245	a-HM035285
*Elymus elymoides*	PI 531606	1	**St**	b-AY553249	a-HM035246	a2-AY010992
			**H**	a-AY553244	c-HM035247	d2-AY010965
*Elymus glaucus*	RJMG 130	4	**St**	-	-	a-AY01097
			**H**	-	-	b-AY010966
*Elymus glaucus*	W6 10215	6	**St**	b-AY553250	NR^5^	b-AY010980
			**H**	e-HM035316	a-HM035248	a-AY010967
*Elymus hystrix*	Barkworth 97–87	1	**St**	b-AY553251	NR	a-AY010982
			**H**	a-AY553245	a-HM035249	d-HM035286
*Elymus lanceolatus*	W6 14220	1	**St**	-	c-HM035250	a-AY010993
						d-AY010984
			**H**	-	a-HM035251	aa-AY010969
*Elymus lanceolatus*	W6 14218	2	**St**	-	a-HM035252	c-AY010985
						d-AY010994
			**H**	-	h-HM035253	a-AY010970
*Elymus lanceolatus*	PI 531623	3	**St**	b-AY553252	-	-
			**H**	a-AY553246	-	-
*Elymus riparius*	RJMG 160	1	**St**	-	a-HM035254	NR
			**H**	-	b-HM035255	a-AY010971
*Elymus trachycaulus*	PI 372500	1	**St**	e-HM035317	a-HM035256	b1-AY010986
			**H**	a-HM035318	b-HM035257	a1-AY010972
*Elymus trachycaulus*	PI 452446	3	**St**	-	-	d-AY010987
			**H**	-	-	a-AY010973
						b-AY010974
*Elymus virginicus*	RJMG 161	4	**St**	NR	NR	d-AY010995
			**H**	a-HM035319	a-HM035258	a-AY010975
*Elymus virginicus*	RJMG 168	9	**St**	d-HM035320	a-HM035259	a-AY010989
			**H**	a-HM035321	e-HM035260	b-AY010976
*Elymus wawawaiensis*	PI 285272	1	**St**	-	a-HM035261	c-AY010996
			**H**	-	d-HM035262	a-AY010977
*Elymus wawawaiensis*	PI 598812	3	**St**	b-AY553253	c-HM035263	a-AY010990
						b-AY010997
			**H**	a-AY553247	b-HM035264	d-AY010978

1 Plant introduction (PI) and W6 numbers were assigned by the USDA; RJMG and Barkworth accessions are from the first author and M. Barkworth, respectively. The *E. trachycaulus* accessions were collected in Canada; all others are from the United States. Voucher specimens are at ID.

2 Numbers (#) distinguish individuals within species on Figures 1–3, S1.

3 Letters identify cloned sequences from within each individual on Figures 1–3, S1.

4 Dashes indicate individuals that were not sampled for the corresponding gene.

5 NR: not recovered for a gene/individual.

6,7 AY5532## accessions: [56]; AY0109## accessions: [29]; all others are new.

Three single- or low-copy nuclear genes were amplified from each *Elymus* individual, and 8–24 clones per gene per individual were checked; the goal was to obtain copies from the **St** and **H** genomes from each. For each gene, the cloned sequences from *Elymus* were analyzed with previously-published sequences from a reasonably broad sample of the tribe's known genomic diversity, including representatives of 15 monogenomic genera (Table 3). These include the donor of the **St** genome (*Pseudoroegneria*), and of the genomes known to co-occur with **St** in allopolyploid nuclei: *Hordeum* (**H**), *Agropyron* (**P**), *Australopyrum* (**W**), *Psathyrostachys* (**Ns**; this genome is represented by tetraploid *Leymus* Hochst. in the pepC data set), and *Thinopyrum* (**J** and/or **E**). Additional monogenomic genera include *Aegilops* L., *Crithopsis* Jaub. & Spach (except for pepC), *Dasypyrum* (Coss. & Durieu) T.Durand, *Eremopyrum* (Ledeb.) Jaub. & Spach, *Henrardia* C.E.Hubb. (except for pepC), *Heteranthelium* Hochst. ex Jaub. & Spach, *Peridictyon* Seberg, Fred., & Baden, *Secale* L., *Taeniatherum* Nevski, and *Triticum*. The sample includes most of the monogenomic genera accepted in genome-based classifications of the Triticeae (e.g., [35], [36]). All three gene trees were rooted with a representative of *Bromus* L.; Bromeae and Triticeae have repeatedly been shown to form a single clade, with Bromeae as either sister or paraphyletic to a monophyletic Triticeae [57], [58].

**Table 3:** Non-allopolyploid representatives of the Triticeae.

Species name	Accession	#	pepC	β-amylase	GBSSI
*Aegilops bicornis* (Forsskål) Jaub. & Spach.	Morrison s.n.		-	4AY821686	-
*Aegilops caudata* L.	G 758		-	4AY821687	6AF079262
				4AY821688	
				4AY821689	
*Aegilops comosa* Sibth. & Smith	G 602		1AY553236	4AY821690	-
				4AY821696	
*Aegilops speltoides* Tausch	Morrison s.n.		-	-	6AF079267
*Aegilops tauschii* Coss.	Morrison s.n.		-	4AY821695	6AF079268
*Aegilops uniaristata* Vis.	G 1297		-	4AY821691	6AF079270
*Agropyron cristatum* (L.) Gaertn.	PI 279802	1	1AY553237	4AY821697	6AF079271
*Agropyron cristatum*	PI 281862	2	-	-	7AY011002
*Agropyron mongolicum* Keng	D 2774		-	-	7AY011003
*Australopyrum retrofractum* (Vickery) Á.Löve	PI 533013		-	4AY821692	6AF079272
*Australopyrum velutinum* (Nees) B.K.Simon	D 2873–2878		1AY553238	4AY821693	7AY011004
*Crithopsis delileana* (Schult.) Rosch.	H 5562		-	4AY821694	5GQ847707
*Dasypyrum villosum* (L.) Candargy	PI 251478	1	-	4AY821698	6AF079274
*Dasypyrum villosum*	PI 470279	2	-	4AY821699	-
*Dasypyrum villosum*	D 2990	3	1AY553240	-	-
*Eremopyrum bonaepartis* (Spreng.) Nevski	H 5554		-	4AY821700	7AY011005
*Eremopyrum distans* (C.Koch) Nevski	H 5552		-	4AY821701	7AY011006
*Eremopyrum orientale* (L.) Jaub. & Spach	H 5555		1AY553254	4AY821702	7AY011007
*Henrardia persica* (Boiss.) C.E.Hubb.	H 5556		-	4AY821703	6AF079276
*Heteranthelium piliferum* (Banks & Sol.) Hochst.	PI 402352		1AY553255	4AY821704	6AF079277
*Hordeum bogdanii* Wilensky	PI 531762^11^	1	-	5GQ847675	2EU282316
*Hordeum bogdanii*	PI 531760^11^	2	2EU282293	2EU282255	2EU282317
*Hordeum brevisubulatum* (Trin.) Link	PI 401387	1	-	4AY821705	7AY010961
				4AY821712	
*Hordeum brevisubulatum*	PI 401390	2	-	4AY821713	7AY010964
*Hordeum bulbosum* L.	PI 440417	1	2EU282294	4AY821706	7AY010962
			2EU282295		
			2EU282296		
*Hordeum californicum* Covas & Stebbins	MA-138-1-40	1	1AY553256	4AY821707	6AF079273
*Hordeum chilense* Roem. & Schult.	PI 531781^11^	1	2EU282297	-	2EU282318
*Hordeum jubatum* L.	RJMG 106^11^	1	1AY553257	4AY821708	7AY010963
			10HM035287	4AY821709	10HM035265
*Hordeum jubatum*	RJMG 134^11^	2	10HM035288	4AY821710	10HM035266
			10HM035289	4AY821711	10HM035267
*Hordeum marinum* Huds.	PI 304346^11^	1	1AY553258	2EU282256	7AY010959
				2EU282257	
*Hordeum marinum*	PI 304347^11^	3	2EU282298	2EU282258	2EU282319
*Hordeum murinum* L.	PI 247054^11^	1	2EU282299	2EU282259	2EU282320
			2EU282300		
*Hordeum murinum*	CIho 15683^11^	2	1AY553259	2EU282260	7AY010960
*Hordeum pusillum* Nutt.	CIho 15654^11^		2EU282301	2EU282261	2EU282321
*Hordeum stenostachys* Godr.	PI 531791^11^	1	2EU282302	2EU282262	2EU282322
*Hordeum vulgare* L.		1	-	-	8X07931
*Hordeum vulgare*	RJMG 107^11^	2	1AY553260	2EU282263	-
*Leymus racemosus* ssp. *sabulosus* (M.Bieb.) Tzvelev	PI 531813		1AY553261	-	-
*Peridictyon sanctum* (Janka) Seberg, Fred., & Baden	KJ 248		1AY553262	4AY821714	6AF079278
*Psathyrostachys fragilis* (Boiss.) Nevski	PI 343192		-	4AY821715	6AF079279
*Psathyrostachys juncea* (Fisch.) Nevski	PI 206684		-	4AY821716	6AF079280
*Pseudoroegneria libanotica* (Hack.) D.R.Dewey	PI 228391	1	2EU282304	2EU282264	2EU282324
*Pseudoroegneria libanotica*	PI 228392	3	2EU282305	2EU282265	2EU282325
*Pseudoroegneria spicata* (Pursh) Á.Löve subsp. *spicata*	PI 232117	1	-	4AY821717	6AF079281
*Pseudoroegneria spicata* subsp. *inermis* (Scribn. & J.G.Smith) Á.Löve	PI 236681	2	-	4AY821718	7AY010998
*Pseudoroegneria spicata* subsp. *spicata*	PI 610986	3	1AY553263	-	7AY010999
*Pseudoroegneria spicata* subsp. *spicata*	D 2844	4	1AY553264	4AY821719	7AY011000
*Pseudoroegneria spicata* subsp. *spicata*	RJMG 112^11^	6	-	4AY821720	7AY011001
					7AY010991
*Pseudoroegneria stipifolia* (Czern. ex Nevski) Á.Löve	PI 313960	2	2EU282306	2EU282266	-
*Pseudoroegneria stipifolia*	PI 531751	3	2EU282307	4AY821721	-
			2EU282308		
*Pseudoroegneria strigosa* (M.Bieb.) Á.Löve	PI 499637	1	2EU282309	-	2EU282323
			2EU282310		
*Pseudoroegneria strigosa* ssp. *aegilopoides* (Drobow) Á.Löve	PI 531755	2	2EU282311	2EU282267	9AY360823
*Pseudoroegneria tauri* (Boiss. & Balansa) Á.Löve	PI 380652	1	2EU282312	2EU282268	2EU282326
*Pseudoroegneria tauri*	PI 401319	2	2EU282313	-	2EU282327
*Pseudoroegneria tauri*	PI 380644	3	2EU282314	-	-
			2EU282315		
*Secale cereale* L.	Kellogg s.n.		1AY553266	4AY821723	7AY011009
				4AY821724	
*Secale montanum* (C.Presl.) C.Presl.	T 36554		-	4AY821725	-
*Secale montanum*	PI 440654		-	-	6AF079282
*Secale strictum* subsp. *anatolicum* (Boiss.) K.Hammer	PI 206992		1AY553265	4AY821722	7AY011008
*Taeniatherum caput-medusae* (L.) Nevski	PI 208075	1	-	4AY821726	7AY011010
*Taeniatherum caput-medusae*	RJMG 189^11^	2	1AY553268	4AY821727	9AY360847
					9AY360848
*Taeniatherum caput-medusae*	PI 314697^11^	3	-	4AY821728	-
*Taeniatherum caput-medusae*	PI 317475^11^	4	-	4AY821729	-
*Thinopyrum bessarabicum* (Savul. & Rayss) Á.Löve	PI531711		-	4AY821730	6AF079283
*Thinopyrum elongatum* (Host) D.R.Dewey	PI 531719	1	-	4AY821731	6AF079284
*Thinopyrum elongatum*	RJMG 113^11^	2	1AY553269	-	-
*Thinopyrum scirpeum* (C.Presl) D.R.Dewey	PI 531749		-	5GQ847676	7AY011011
*Triticum aestivum* L.			3AJ007705	-	-
*Triticum baeoticum* Boiss.	Morrison s.n.		-	4AY821732	6AF079285
*Triticum monococcum* L.	PI 221413		-	4AY821733	-
*Triticum urartu* Tumanian	Morrison s.n.		-	5GQ847677	6AF079287
*Bromus tectorum* L.	Kellogg s.n.		1AY553239	4AY821734	9AY362757

1–9 From [56], [21], [59], [61],[62],[63],[29],[64],[60], respectively.

10 New to this study.

11 Indicated accessions are deposited at ID; others are at GH.

Nearly all of the sequences from the monogenomic species were previously published in various sources [21], [29], [56], [59]–[64], with a few exceptions as noted in Table 3. Information about the data and taxa can be found therein, but the primary discussions about the characteristics of each marker and data set are: pepC [56]; β-amylase [61]; and GBSSI [29], [63]. Based on studies of grass genomes in crop species, the three nuclear markers appear to be on three different chromosomes (more below). This assumption is tentative because it is based on a small number of grass species, but for this study the three genes are assumed to be unlinked, and to represent independent phylogenetic estimates.

### Molecular methods and alignment

Similar molecular methods were followed for each of the three nuclear gene fragments (detailed protocols are found in the works cited above for each marker). For each *Elymus* individual, three PCR replicates were run per individual, in order to counter the potential effects of PCR drift [65]. PCR products from replicate reactions were combined and cleaned on columns (Qiagen). Cleaned products were cloned into pGEM-T Easy vectors (Promega), and transformed into *E. coli* JM109 competent cells (Promega) following the manufacturer's protocol, except that all reactions were halved. Cloned fragments were amplified directly from white colonies using the same primers as were used for the original PCR, in 30–40 µl reactions with 0.5 units Taq polymerase (Sigma), a 1× concentration of the included Taq buffer, 45–60 nmol MgCl_2_, 6–8 nmol of each nucleotide, and 30–40 pmol of each primer. Amplified fragments were cleaned for sequencing using 1 unit shrimp alkaline phosphatase (USB) and 5 units exonuclease I (USB). Sequencing reaction included 1–3 µl of cleaned product, 2 µl BigDye Terminators v.3.1 (Applied Biosystems), and 3.2 pmol primer in a 10 µl volume. Reactions were run on an ABI Prism 377 (our lab) or ABI 3730 DNA Analyzer (Pritzker Lab, Field Museum of Natural History). Between 8 and 24 cloned PCR products from each individual were screened with a single sequencing primer, yielding a single-stranded partial sequence of about 600 basepairs. We examined these preliminary sequences to identify the two homoeologous sequence types (**St** and **H**) that we expected to find within each tetraploid individual. Representative clones of each were fully sequenced on both strands and added to the data set. If either homoeologous copy was missing from an initial sample of 8–12 clones, the corresponding gene from that individual was re-amplified and cloned, and 12 additional sequences were screened. We also included distinct, same-genome alleles from within individuals when they were encountered, although this was not our main goal. Based on the 600-basepair preliminary sequences, same-genome sequences that differed by more than three basepair substitutions were fully sequenced and added to the data set. The three-basepair threshold was arbitrary, but we reasoned that it was large enough to reveal distinct alleles rather than Taq errors.

PCR amplification of intra-individual variants can yield chimeric sequences [66]–[68]. A small number of recombinants were identified by inspection of alignments prior to phylogenetic analysis; such sequences are often visible because the **St** and **H** variants have length differences in some of the introns. A few more were identified following closer examination of sequences on long branches in preliminary maximum parsimony analyses. Such sequences were confirmed as recombinant by inspection or if, when they were divided at the presumed point of recombination and analyzed as separate sequences, one portion was phylogenetically **St**-like and the other was **H**-like. Chimeric sequences were interpreted as PCR artifacts and removed from the analyses.

The pepC gene is a member of a three-copy family in grasses [69]; the sequences used here appear to be homologous to the widely-expressed housekeeping copy. Based on the location of similar sequences in the rice genome (Genbank AP005781 and AP005802) and a comparative grass genome map [70], this gene copy is assumed to be on the Triticeae group 5 homoeologous chromosomes. The original Triticeae pepC data set [56] combined two fragments designated region 1 (approximately 1 kb; Genbank AY553236–AY553269) and region 2 (approximately 600 bp; Genbank AY548399–548432); the present data set includes just region 1 sequences. The 1100-bp PCR products obtained using primers 467F(1) and 1672R(2) [56] include partial exons 1 and 2, along with the intervening intron, which is approximately 1000 bp long. The intron exhibits considerable length variation, including insertion and excision of transposons [21]. Most length variation could be accommodated by manually adjusting the alignment (File S1). An ambiguous region of the alignment consisting mainly of short runs of C and/or T (positions 67–109), and two regions affected by transposon activity (690–771 and 1035–1119), were excluded from the analysis.

The β-amylase genes form a small family in the Triticeae, with several copies expressed in the endosperm and one that is ubiquitously expressed [71]. Based on sequence similarity, the sequences used here appear to represent the ubiquitously-expressed copy; this copy has been mapped to the Triticeae group 2 homoeologous chromosomes [72]. The 1400-bp β-amylase PCR products were obtained using primers 2a-for and 5a-bac [61], and include partial exons 2 and 5, complete exons 3 and 4, and introns 2–4, which are about 250, 100, and 400 bp in length, respectively. The β-amylase alignment (File S2) was generally straightforward; most length differences were easy to interpret. One ambiguous simple sequence region (positions 553–570) and two regions corresponding to *Stowaway*-like transposon activity in some sequences (positions 635–765 and 1475–1641) [73] were excluded from the analyses.

The GBSSI PCR products were obtained using the F-for and M-bac primers [63], which amplify an approximately 1300-bp fragment that includes partial exons 9 and 14, exons 10–13, and introns 9–13, which are about 100 bp each. The putatively single-copy GBSSI gene maps to the Triticeae group 7 homoeologous chromosomes [70], [74], or to a portion of chromosome 4 translocated from, and thus homoeologous to, the group 7 chromosomes [70], [75]. The GBSSI alignment (File S3) is generally straightforward in spite of numerous small insertions and deletions in the introns. Three ambiguously-aligned regions (positions 910–1020, 1138–1231, and 1383–1515) were excluded from the phylogenetic analyses.

### Phylogenetic analyses

Prior to phylogenetic analyses, 16 nested models of sequence evolution [76]–[78] were examined for each data set using preliminary maximum parsimony trees, and the resulting maximum-likelihood (ML) scores were compared using a likelihood ratio test [78]–[81]. For each data set, the general time-reversible (GTR) substitution model [82], [83] led to a large and significant increase in score compared to the Jukes-Cantor [84], Kimura two-parameter [85], and Hasegawa-Kishino-Yano [86] models, as did the addition of a gamma (Γ) distribution with shape parameter α to model among-site rate variation [87]. Adding an invariable sites (I) parameter [86] to the GTR+Γ model led to a non-significant increase in the pepC and β-amylase scores, and a significant increase in the GBSSI score. Therefore, the GTR+Γ model was used for the ML analysis of the pepC and β-amylase data, and the GTR+I+Γ model was used for the analyses of the GBSSI data sets.

All ML analyses were run using the Mac OS X UNIX version of GARLI v. 0.95 [88]. Following the recommendations of the author, runs were set for an unlimited number of generations, and automatic termination following 10,000 generations without a significant (lnL increase of 0.01) topology change. For each data set, thirty analyses were run with random starting tree topologies, and the tree with the best score was used to display the gene tree. Branch support (BS) for each tree was estimated based on 100 ML bootstrap replicates in GARLI with searches as above, except that the stopping criterion was lowered to 5,000 generations without a significant topology change. Bootstrap values≥50% were recorded on the best ML trees.

## Results

### General

On all three gene trees (Figures 1–3) the *Elymus* sequences fall into two main groups, with *Pseudoroegneria* and with *Hordeum*. The two distinct *Elymus* groups are interpreted as **St** and **H** sequences, respectively, derived from *Pseudoroegneria* and *Hordeum* progenitors. Nearly all of the *Elymus* individuals yield sequences in both of the main clades, with the following exceptions: for pepC, only an **H** sequence was recovered from *E. virginicus* 4 and *E. mutabilis* 1; for β-amylase, only an **H** sequence was recovered from *E. glaucus* 6, *E. hystrix* 1, *E. virginicus* 4, and *E. sibiricus* 1; and for GBSSI, only an **H** sequence was recovered from *E. riparius* 1. No individuals lack a sequence type in all three data sets, and only *Elymus virginicus* 4 is missing a sequence type for two of the three genes. There are cases where individual plants show within-genome (presumably allelic) variation, and these cases are used where possible to shed light on the evolutionary history of the **StStHH** *Elymus* group. However, the study was designed primarily to capture intergenomic variation within individuals (i.e., **St** vs. **H**) rather than allelic variation, so patterns of intra-individual variation within genomes are probably more widespread than the data show.

**Figure 2 pone-0010989-g002:**
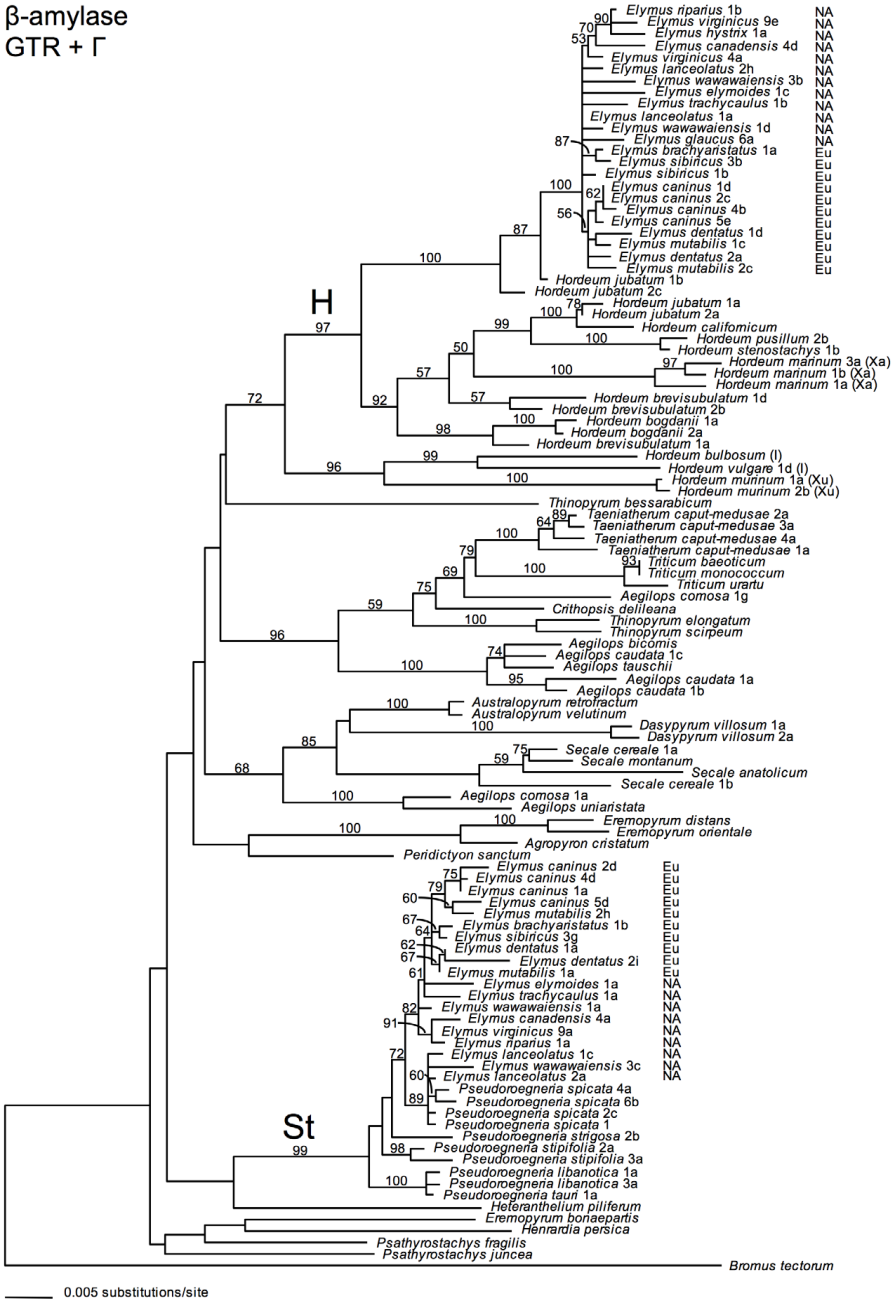
β-amylase gene tree. The best-scoring ML tree was selected from 30 GARLI analyses of the β-amylase sequence data set under a GTR+Γ model of sequence evolution. Numbers above branches show ML bootstrap support ≥50%. “NA” and “Eu” distinguish North American and Eurasian *Elymus* species, respectively. Where applicable, numbers following taxon names distinguish individuals within species, and are consistent among Figures 1–3, S1. Letters following these numbers designate cloned sequences from within individuals, and are specific to each gene tree.

**Figure 3:**
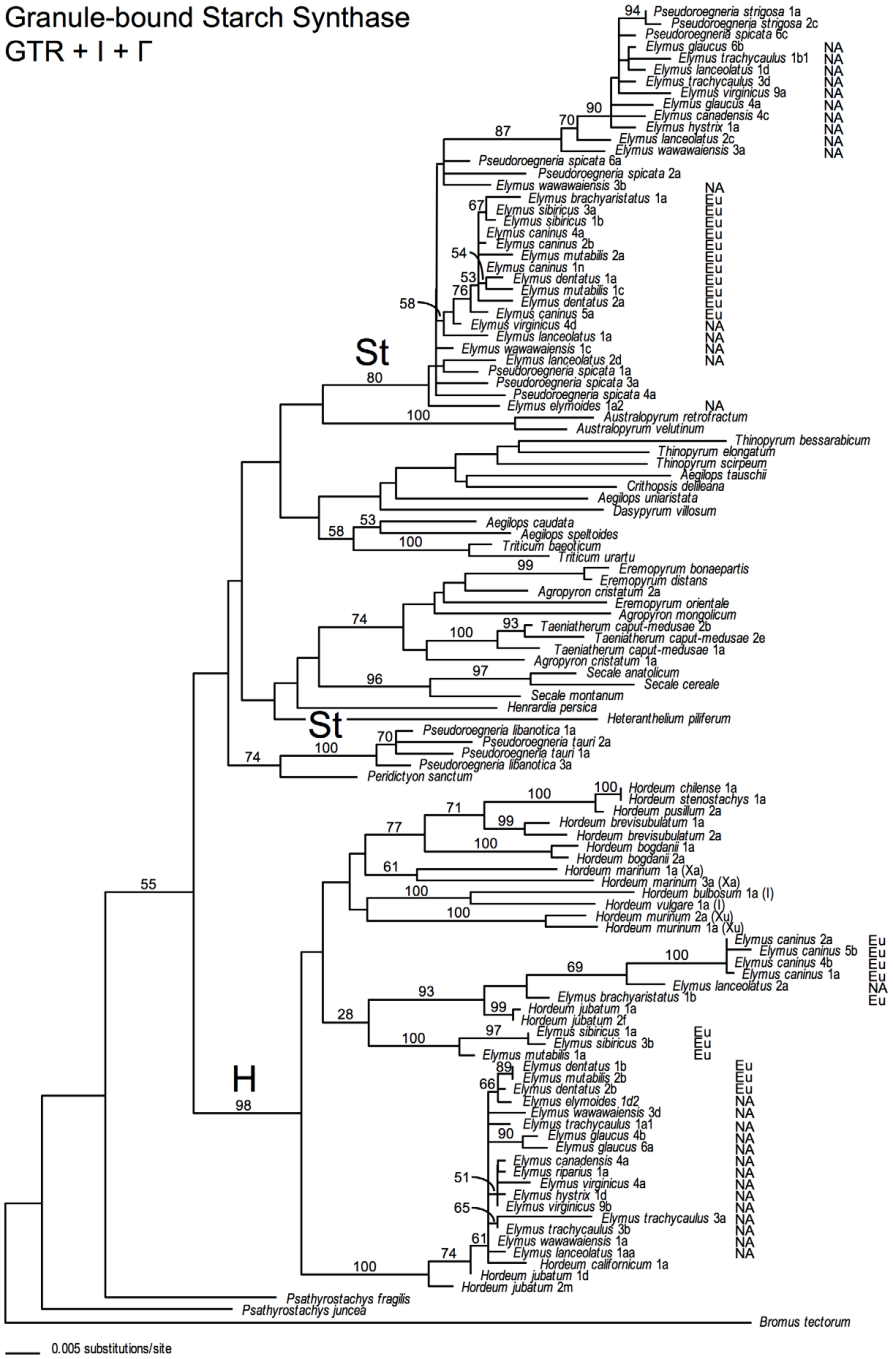
Granule-bound starch synthase gene tree. The best-scoring ML tree was selected from 30 GARLI analyses of the GBSSI sequence data set under a GTR+I+Γ model of sequence evolution. Numbers above branches show ML bootstrap support ≥50%; the 28% value is mentioned in the text. “NA” and “Eu” distinguish North American and Eurasian *Elymus* species, respectively. Where applicable, numbers following taxon names distinguish individuals within species, and are consistent among Figures 1–3, S1. Letters following these numbers designate cloned sequences from within individuals, and are shared by Figures 3 and S1.

There are several cases of apparent β-amylase homeolog silencing in *Elymus*, inferred from exon sequences. Of the Eurasian species, two of the four *E. caninus* individuals have a stop codon in exon 2 of their **St**-genome copies (clones 2d and 4d). Silent copies are more widespread among the North American individuals, and all involve the **H**-genome copy. The *E. hystrix* 1a, *E. riparius* 1b, and *E. virginicus* 9e clones share a 2-basepair deletion in exon 2, and *E. hystrix* 1a has a second, single-basepair deletion in exon 4; the *E. elymoides* 1c and *E. glaucus* 6a clones each have a unique, single-basepair deletion in exon 2; and *E. wawawaiensis* 3b has a stop codon in exon 4. Note that for two individuals (*E. glaucus* 6 and *E. hystrix* 1), no functional β-amylase copy was recovered; in both cases, the **St** copy was not present among the sequenced clones, and the **H** copy includes a frame-shifting deletion.

### The pepC phylogeny

The **St**-genome sequences of *Pseudoroegneria* and *Elymus* form a well-supported clade (100% BS; Figure 1). The *Elymus* **St** sequences show little diversity overall, and are most closely related to the only native North American *Pseudoroegneria* species, *P. spicata* (85% BS), from which they show very little divergence. The only phylogenetic structure within the clade groups the two *E. sibiricus* sequences with *E. brachyaristatus* (70% BS), and one of the two *E. dentatus* sequences with *E. mutabilis* (84% BS). There is no phylogenetic distinction between the North American and Eurasian species.

The remaining *Elymus* pepC sequences represent the **H**-genome, and form a clade with part of *Hordeum* (Figure 1). The *Elymus* **H**-genome sequences, like the **St** sequences, show little diversity. They form a clade (78% BS) with sequences from *H. californicum*, a native North American diploid species, and with *H. jubatum*, a North American allotetraploid whose ITS sequences were derived from *H. californicum* and *H. roshevitzii* [89]. This clade includes three subgroups: most of *Elymus* along with *H. californicum* and one of the *H. jubatum* genomes (77% BS); the four remaining *Elymus* sequences (74% BS); and the second *H. jubatum* genome (95% BS). The four individuals in the smaller *Elymus* clade (*E. mutabilis* 1 and 2, *E. dentatus* 1, and *E. caninus* 2) are also represented in the larger **H** clade; thus, the small clade appears to reveal allelic variation in the **H** genome. The three species in this clade are Eurasian natives, so the allele may be restricted to Europe, though more intensive sampling would be required to support this.

### The β-amylase phylogeny

Within the **St**-genome clade on the β-amylase tree (Figure 2; 99% BS), the *Elymus* sequences are again most closely related to *P. spicata*, though the support for this relationship is only moderate (72% BS). Compared to the pepC tree, there is more phylogenetic structure among the β-amylase **St** sequences of *Elymus* and *P. spicata*, including weak (64% BS) support for a Eurasian species clade within a paraphyletic North American *Pseudoroegneria*/*Elymus* assemblage. Intraspecific sampling is limited, but within the Eurasian clade, the **St** sequences representing *E. dentatus* 1 and 2 form a monophyletic group (62% BS), while those from *E. caninus* and from *E. mutabilis* do not. The **St** sequences representing the North American species *E. wawawaiensis* are non-monophyletic, and those from *E. lanceolatus* 1 and 2 are unresolved; the remaining species only have a single representative on the tree.

In contrast to the pepC tree, the **H** sequences from *Elymus* do not group with the diploid *H. californicum* on the β-amylase tree, but only with one of the *H. jubatum* genomes (100% BS). The *H. californicum* sequence forms a clade with the other *H. jubatum* genome (100% BS) within a large, multi-species *Hordeum* clade (92% BS) sister to (97% BS) the *Elymus*/*H. jubatum* clade. There is much less resolution among the *Elymus* **H** sequences than among the **St** sequences. The few relationships with >50% BS support are within-continent groups, but there is no suggestion of a Eurasian species clade, as there is in the **St**-sequence group.

### The GBSSI phylogeny

The structure of the **St**-genome clade on the GBSSI tree differs from those on the other two trees. The group includes a paraphyletic “core” assemblage on short branches, in which the *Elymus* sequences are similar to those from five *P. spicata* individuals (1, 2, 3, 4, and 6). Within this assemblage, the Eurasian *Elymus* sequences form a moderately supported clade (76% BS) derived from within a paraphyletic group that includes *P. spicata* and six sequences from four North American *Elymus* species. This pattern by itself is similar to that in the β-amylase **St** clade, and is suggestive of a single origin of Eurasian species from within a paraphyletic group of North American *Elymus* and *Pseudoroegneria* species. The GBSSI **St** clade, however, is unique in having a separate subclade (87% BS) on a relatively long branch nested within the core group. The subclade includes the second of two sequences from *P. spicata* 6, the remaining ten North American *Elymus* sequences, and, surprisingly, two Chinese accessions of *P. strigosa*. Like *P. spicata* 6, three *Elymus* individuals have gene copies in both the paraphyletic assemblage of short branches, and in the long-branched subclade: *E. lanceolatus* 1 (clones a and d) and 2 (d and c), and *E. wawawaiensis* 3 (b and a). At the species level, *E. virginicus* is also represented in both groups, with individual 4 separated from individual 9. Another unique feature of the GBSSI tree is the placement of *P. libanotica* and *P. tauri* far outside of the main **St**-sequence clade, though their position within the tree is not convincingly resolved. The placement of these sequences as a possible result of diploid-level introgression, and their contribution to some of the species in a different group of *Elymus* tetraploids (genomes **StStYY**) is discussed elsewhere [62]. Based on the present sample, however, these species play no role in the evolution of the **StStHH** *Elymus* tetraploids.

The GBSSI **H**-genome clade, like the **St**-genome clade, is more complex than its counterparts on the pepC and β-amylase trees. All but one of the North American *Elymus* sequences form a well-supported clade (100% BS), along with *H. californicum*, one of the tetraploid *H. jubatum* genomes, and two Eurasian species (*E. dentatus* 1 and 2, and *E. mutabilis* 2). The remaining, mostly-Eurasian *Elymus* sequences form a very weak (28% BS) group with much greater sequence diversity. This assemblage includes a small clade of sequences from *E. sibiricus* 1 and 3 and *E. mutabilis* 1 (100% BS), and a larger clade (93% BS) with the second *H. jubatum* genome, two Eurasian *Elymus* species (*E. brachyaristatus* and *E. caninus*), and one accession of the North American species *E. lanceolatus*. The high diversity among the GBSSI **H**-genome sequences, compared to those on the pepC and β-amylase trees, was unexpected enough to raise suspicion about previously undetected alignment artifacts in the introns. There is considerable length variation in the GBSSI introns, so we ruled out the possible effects of non-orthologous intron alignment by running a ML analysis of just the **H**-genome clade using the exons only, for which the alignment is unambiguous. The resulting tree (Figure S1) has a similar (though less well-resolved) topology, including the same clade of very similar, mostly North American sequences, with the remaining mostly-Eurasian sequences forming a weak group of long branches outside of the main *Elymus* clade.

## Discussion

### The **StStHH** genome configuration

Our initial assumption that the *Elymus* species included here are **StStHH**-genome tetraploids was based on the results of numerous studies of meiotic chromosome pairing (e.g., [54] and references therein). As expected, all three gene trees unequivocally support the *Pseudoroegneria* + *Hordeum* origin of the sampled *Elymus* species, in agreement with an earlier analysis of RPB2 gene sequences [90]. Nearly all of the *Elymus* individuals have two distinct copies of all three genes, in clades with *Pseudoroegneria* and *Hordeum*. No individuals are missing either copy of all three genes; thus, the occasional missing copies from a few individuals might represent sampling artifacts, copy loss, or unique changes in primer sites, but do not suggest that either genome is absent altogether.

### The pepC and β-amylase trees

The pepC and β-amylase trees are in general agreement with regard to the relationships among the *Elymus* species. Both show the North American and Eurasian species to be very closely related, and neither support independent origins for the two geographic groups. The **St**- and **H**-genome clades on both trees support a North American origin for the **StStHH** *Elymus* tetraploids. Of the *Pseudoroegneria* and *Hordeum* species included in the analysis, those most closely related to *Elymus* are North American species. Furthermore, within the **St**-sequence clade on the β-amylase tree, the Eurasian *Elymus* species form a clade (albeit weakly supported) within a broader paraphyletic assemblage of North American *Elymus* and *Pseudoroegneria* sequences. Evidence for a single, North American origin of the **StStHH** tetraploids is at odds with the earlier suggestion that the North American and Eurasian species arose separately, based on limited karyotype [91] and isoenzyme data [92], [93]. More recently, separate origins were moderately well-supported in a phylogenetic analysis of a nuclear RNA polymerase II (RPB2) gene [90]; **H**-genome RPB2 sequences separated *Elymus* into largely American and Eurasian subclades with, respectively, American and Eurasian *Hordeum* species. The RPB2 **St**-genome sequences did not reveal a geographic pattern, and were ambiguous with regard to whether the closest *Pseudoroegneria* species was North American or Eurasian, placing one accession each of *P. spicata* and *P. stipifolia* within *Elymus*, and a second accession of *P. spicata* outside of the *Elymus* clade on a very long branch. (While all of our trees implicate *P. spicata* as a potential donor to the **StStHH** tetraploids, none point to *P. stipifolia*.)

The pepC and β-amylase trees do differ with respect to the relationships between *Elymus* and *Hordeum*; specifically, the possible roles of diploid *H. californicum* and allotetraploid *H. jubatum* in the origin of **StStHH** *Elymus*. On the pepC tree, the **H**-genome sequences of *Elymus* are grouped with, and very similar to, the North American diploid *H. californicum*, and with both genomes of the allotetraploid *H. jubatum*. This is consistent with the results of an earlier study of repetitive DNA sequences [94], and one based on starch synthase data [29], both of which suggested *H. californicum* as a possible **H**-genome donor to *Elymus*. However, in contrast to the pepC tree, the β-amylase tree does not place *Elymus* with *H. californicum*, but instead with one of the *H. jubatum* genomes, while *H. californicum* is grouped with the other *H. jubatum* genome in a separate, multi-species *Hordeum* clade. Together, these trees and the differences between them suggest that a tetraploid similar to *H. jubatum* might have been involved in the history of *Elymus*, whether through past introgression among the *Elymus*, *H. californicum*, and *H. jubatum* lineages, or through a direct contribution from a tetraploid *H. jubatum*-like species to *Elymus*. Direct involvement of an *H. jubatum*-like ancestor would have led to the simultaneous introduction of both of its homoelogous **H** genomes, and in a successfully diploidized **StStHH** tetraploid, they would then behave as homologous alleles. Thus, depending on changes in allele frequency through time, *Elymus* might exhibit one or the other, or both, of the *H. jubatum*-like homoeologs.

The relationships among **H**-genome sequences could, instead, reflect introgression following tetraploid *Elymus* formation. It is impossible to trace a precise sequence of events, but we can envision scenarios consistent with the data. For example, if *H. californicum* was, in fact, the **H**-genome donor to **StStHH** *Elymus*, as suggested by the pepC tree, then *H. californicum* could be “misplaced” on the β-amylase tree, having acquired its β-amylase gene copy through introgression after the formation of *Elymus*. Alternatively, *Elymus*'s placement on the β-amylase tree, far from *H. californicum*, might indicate that it was *Elymus*'s β-amylase gene that was acquired through introgression; its close relationship to the second genome of *H. jubatum* indicates that species as a potential source. Additional samples of *H. californicum* and *H. jubatum* might support or refute our hypotheses, or suggest other conceivable scenarios, but in any case, it appears that *H. jubatum* was involved at some stage in the history of **StStHH** *Elymus*.

### The GBSSI tree suggests further introgression or lineage sorting

If *Elymus* relationships are in some ways similar on the pepC and β-amylase trees, and relatively straightforward to interpret, the GBSSI results complicate the interpretation. In the **St** sequence clade, the paraphyletic “core” group of very similar sequences is, by itself, reminiscent of the pattern on the β-amylase tree, with the Eurasian species forming a moderately-supported clade within a paraphyletic group of North American *Elymus* and *Pseudoroegneria* species. This group, when considered alone, supports a North American origin of the **StStHH** tetraploid group from a *P. spicata*-like ancestor. However, the long-branched clade that arises from within the core group is unique to the GBSSI tree. It includes sequences from several North American *Elymus* individuals, some of which are also represented in the core group, and two Chinese accessions of a Eurasian *Pseudoroegneria* species, *P. strigosa*. The clade also includes one *P. spicata* sequence; the same individual (#6) has a second sequence in the core group with the rest of *P. spicata*. The dual placement of several individuals in both the core group and in the derived clade could reveal gene duplication. However, such a duplication event (at the base of the “**St**” clade; Figure 3) should also be evident in *P. strigosa* and in the Eurasian *Elymus* species, unless we postulate at least two subsequent, independent paralog losses. Thus, a more parsimonious explanation is that the relationships among GBSSI sequences from *P. spicata*, *P. strigosa*, and *Elymus* result from either past gene exchange or from the maintenance of a shared ancestral polymorphism. Introgression of a *P. strigosa*-like GBSSI allele into North America could explain the close relationship between *P. strigosa* and some of the sequences from *P. spicata* and North American *Elymus*, though the exact sequence of events is not clear from the present sample. The *P. strigosa* allele might have been introduced to North America through hybridization between *P. spicata* and *P. strigosa*, and then passed from *P. spicata* to North American *Elymus* through hybridization, or through formation of new **StStHH** tetraploids and subsequent hybridization among tetraploid lineages. The allele might have been introduced directly into *Elymus* through hybridization with a tetraploid (**StStStSt**) accession of *P. strigosa*. Given the possible Eurasian origin of the allele, we could also postulate an introduction via Eurasian *Elymus* tetraploids, but so far, none of the sampled Eurasian **StStHH** *Elymus* species have alleles in this clade. Alternatively, the *P. spicata* GBSSI polymorphism, including the allele in a close relationship with the *P. strigosa* sequences, could reflect the maintenance of ancestral polymorphism as a result of incomplete lineage sorting. The subsequent introduction of both alleles from *P. spicata* into North American *Elymus* is consistent with the placement of *Elymus* alleles with both *P. strigosa* and *P. spicata* on the GBSSI tree. The Eurasian *Elymus* species lack the polymorphism; assuming this is not merely a sampling artifact, it appears that the *P. strigosa*-like allele was either never introduced into the Eurasian group, or that it was subsequently lost.

In the GBSSI **H**-clade, there is, again, a “core” group of very similar sequences (though monophyletic in this case) that includes most of the North American and a few Eurasian *Elymus* sequences with *H. californicum* and one genome of the tetraploid *H. jubatum*. The relationships among the *Elymus* sequences in the core clade once again suggest a North American origin, with a clade of Eurasian sequences nested within a paraphyletic North American *Elymus* and *Hordeum* group. However, the arrangement of *Elymus* sequences outside of the core clade bears no resemblance to either of the other trees, or to the **St**-genome clade on this tree. These species are primarily Eurasian, except for one of the two accessions of *E. lanceolatus*. The only *Hordeum* sequences loosely associated with these *Elymus* sequences represent the second genome from the tetraploid *H. jubatum*; this provides some additional support that *H. jubatum* was somehow involved in the history of the **StStHH** *Elymus* species. This interpretation is not entirely satisfying, however, because the pattern is so unlike that on either of the other trees; these *Elymus* species are unexpectedly divergent from *H. jubatum* and from one another. Thus, there is no clear indication of a donor-recipient relationship among the sequences outside the core clade, whether from introgression or otherwise, so it is difficult to speculate on processes that might explain the topology of **H**-genome clade on the GBSSI tree. Consideration of the earlier RPB2 analysis [90] further complicates the interpretation. The Eurasian and American **H**-genome clades uncovered in that study were grouped with American and Eurasian representatives of *Hordeum* – *H. stenostachys* and *H. bogdanii*, respectively – but these were the only two *Hordeum* species included in the RPB2 analysis, so a potential role of *H. californicum* and *H. jubatum* in the origin of *Elymus* was not assessed.

### Summary and Conclusion

Our first goal was to test the hypothesis that the North American and Eurasian *Elymus* species arose independently; this was suggested by allozyme [92], [93] and cytological [91] studies, and more explicitly supported by a recent molecular phylogenetic analysis of RPB2 [90]. However, our data support a single, North American origin. In the studies using cytological and allozyme data, issues of small sample size or the interpretation of complicated results could be invoked to explain away the contradiction, but the RPB2 results are more difficult to square with ours. We stand by the conclusion of a single origin because none of our three trees support separate origins. However, there are clearly other processes in play beyond the number of **StStHH** origins, and we have only four nuclear gene trees (including RPB2) in consideration, so additional trees could tip the interpretation in another direction. Our second goal, the identification of possible progenitors within *Pseudoroegneria* and *Hordeum*, yielded reasonably consistent results. *Pseudoroegneria spicata* is supported as the most likely **St**-genome donor among the species included on all of our trees (albeit with complications involving introgression or incomplete lineage sorting on the GBSSI tree), and as a possible progenitor on the RPB2 tree. *Hordeum californicum* and/or its tetraploid derivative *H. jubatum* are suggested as **H**-genome donors to *Elymus* on all three of our trees; past interactions among these species remain to be clarified with more targeted sampling of these species and their closest relatives within *Hordeum*, and additional gene trees. Finally, our third goal was to identify reticulate patterns beyond allopolyploidy. Examination of the GBSSI tree relative to the others revealed a case of introgression or incomplete lineage sorting, revealed by the discovery of *P. strigosa*-like **St**-genome allele in North American *Elymus* and *Pseudoroegneria*, and a more confusing situation involving the GBSSI **H**-genome.

Our attempts to propose evolutionary scenarios to reconcile conflicting patterns among *Elymus* gene trees reveal a general problem with inferring reticulate events from multiple conflicting trees. The interpretation depends heavily on which tree is initially assumed to be closest to the “best” or “true” tree (with or without a clear explanation), after which the differences on the remaining trees are attributed to processes such as introgression or incomplete lineage sorting. In other words, the sequence of historical events leading to gene tree conflict is defined by which tree is selected as a reference tree, or the one with which other trees are viewed to be in conflict. (Perhaps the most familiar examples in plants involve conflicts between ITS and cpDNA trees, which are generally interpreted as cpDNA introgression. In that case, the ITS tree is being used as the reference tree, sometimes without explicit justification.) Furthermore, if there are numerous conflicts among trees, a different tree could potentially be selected as the reference tree for each group of conflicting taxa. If many gene trees are available, and one branching pattern is shared by a large majority of them, then it is probably reasonable to interpret the differences on the few conflicting trees with reference to the majority of trees. With just three trees presented here (or four, including RBP2 [90]), and without a clear majority pattern, the distinction between “real” and “conflicting” relationships is not always straightforward. On one hand, for example, the unexpected placement of some *Pseudoroegneria* and *Elymus* **St** sequences with *P. strigosa* on the GBSSI tree, in conflict with the pepC and β-amylase trees, seems like a fairly straightforward case in which the GBSSI gene was affected by introgression or maintenance of an ancestral polymorphism. On the other hand, an assumption that *H. californicum* is the diploid **H**-genome donor to **StStHH** *Elymus* based on the pepC and GBSSI trees, and consequent interpretation of the β-amylase and RPB2 trees in terms of **H**-genome introgression, is more arbitrary. A proposed evolutionary scenario would be quite different if either the β-amylase or the RPB2 tree were assumed to represent the true tree with respect to **H**-genome sequence relationships. In spite of the difficulties with interpreting gene tree conflict as a specific sequence of evolutionary events, such patterns can certainly highlight the importance of such events, pinpoint the taxa involved, and yield hypotheses to be tested with targeted sampling within and among conflicting taxa, and with data from additional nuclear loci.

## Supporting Information

File S1 Sequence data set - phosphoenolpyruvate carboxylase.
(0.15 MB TXT)

File S2 Sequence data set - beta-amylase.
(0.21 MB TXT)

File S3 Sequence data set - granule-bound starch synthase I.
(0.19 MB TXT)

Figure S1 Gene tree based on granule-bound starch synthase exon sequences. The best-scoring ML tree was selected from 30 GARLI analyses of GBSSI exons under a GTR+I+Γ model of sequence evolution. The taxa are the same as in the **H**-genome clade from Figure 3, but the analysis differs in that introns were excluded. Numbers above branches show ML bootstrap support ≥50%. “NA” and “Eu” distinguish North American and Eurasian *Elymus* species, respectively. Numbers following taxon names distinguish individuals within species where applicable, and are consistent among Figures 1–3, S1. Letters following these numbers designate cloned sequences from within individuals, and are shared between Figures 3 and S1.
(0.52 MB TIF)
