# Supplementary figures and images for: Reticulate Evolutionary History of a Complex Group of Grasses: Phylogeny of Elymus StStHH Allotetraploids Based on Three Nuclear Genes
Source: PLoS One. 2010 Jun 9;5(6):e10989. doi: 10.1371/journal.pone.0010989 (PMC2882950; doi:10.1371/journal.pone.0010989)

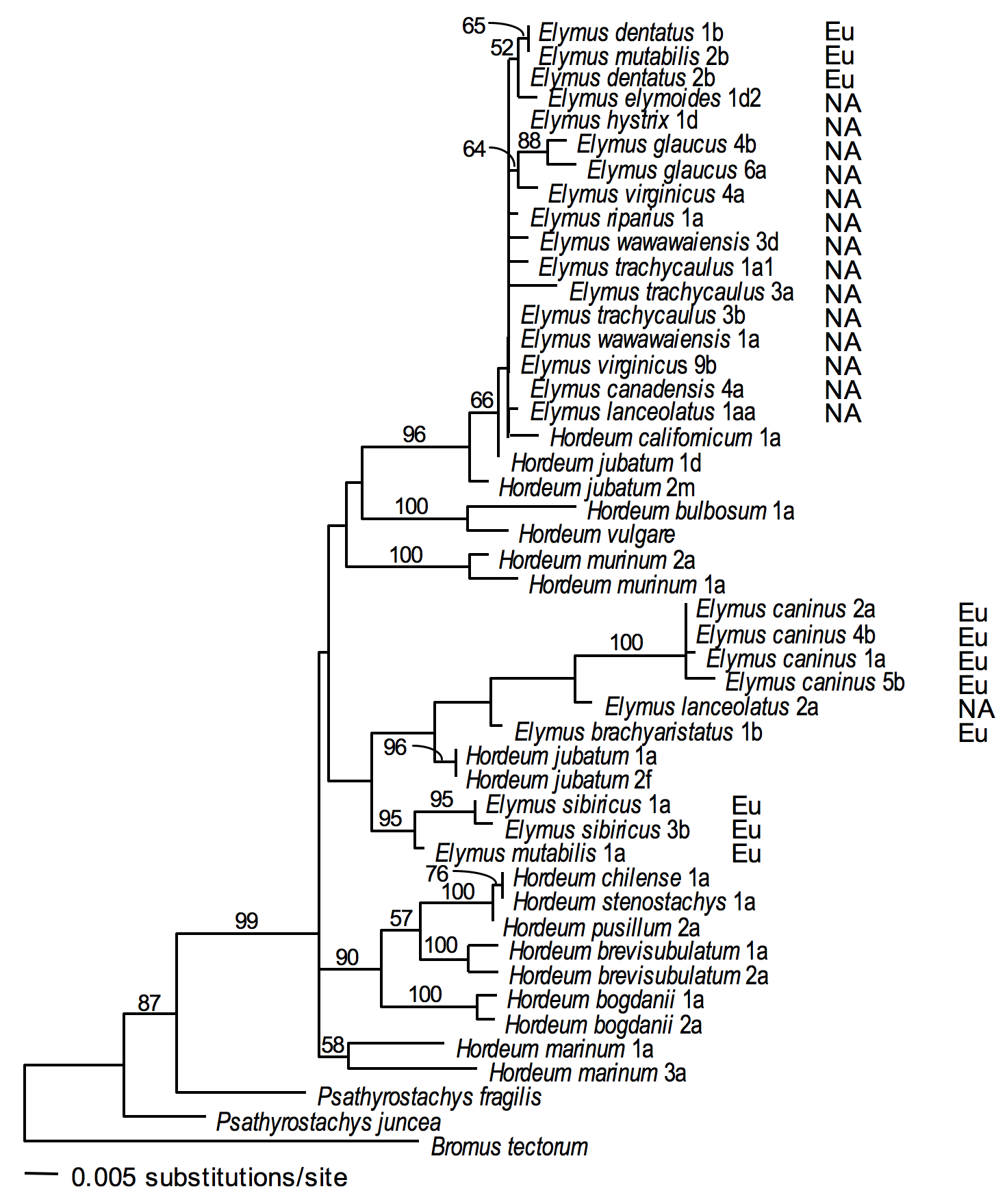

Supplement: Figure S1 — Gene tree based on granule-bound starch synthase exon sequences. The best-scoring ML tree was selected from 30 GARLI analyses of GBSSI exons under a GTR+I+Γ model of sequence evolution. The taxa are the same as in the H-genome clade from Figure 3, but the analysis differs in that introns were excluded. Numbers above branches show ML bootstrap support ≥50%. “NA” and “Eu” distinguish North American and Eurasian Elymus species, respectively. Numbers following taxon names distinguish individuals within species where applicable, and are consistent among Figures 1– 3, S1. Letters following these numbers designate cloned sequences from within individuals, and are shared between Figures 3 and S1. (0.52 MB TIF) [file pone.0010989.s004.tif]
